# Clinical Presentation and Surgical Management of Pediatric Coronary Artery Disease: A Single-Center Retrospective Case Series

**DOI:** 10.7759/cureus.104923

**Published:** 2026-03-09

**Authors:** Khanh Van T Le, Le Duc Tin

**Affiliations:** 1 Department of Pediatric Cardiac Surgery, Cho Ray Hospital, Ho Chi Minh, VNM; 2 Department of Medicine, School of Medicine and Pharmacy, Tra Vinh University, Tra Vinh, VNM; 3 Department of Vascular Surgery, Cho Ray Hospital, Ho Chi Minh, VNM; 4 Department of Thoracic and Vascular Surgery, Nam Can Tho University, Can Tho, VNM

**Keywords:** coronary artery anomalies, coronary artery bypass grafting, coronary vessel surgery, pediatric cardiac surgery, pediatric coronary artery disease

## Abstract

Background

Pediatric coronary artery disease is rare and heterogeneous, with potentially severe clinical consequences. Data from resource-limited settings remains limited. This study aims to describe the clinical presentation and surgical management of pediatric coronary artery disease at a tertiary cardiac surgery center.

Methods

We performed a single-center retrospective case series of pediatric patients who underwent surgical intervention for coronary artery disease between 2018 and 2025. Demographic data, coronary pathology, surgical procedures, and early outcomes were analyzed.

Results

Eight patients (median age 10 years) were included. Fatigue was reported in four patients (50%) and chest pain in two (25%), and most patients had heart failure at presentation. Etiologies included Kawasaki disease-related coronary pathology in three patients (37.5%), anomalous coronary artery origin in three (37.5%, including one case of anomalous left coronary artery from the pulmonary artery (ALCAPA)), coronary artery fistula in two (25%), and tetralogy of Fallot-associated coronary anomalies in two (25%). Eleven surgical procedures were performed, with coronary artery bypass grafting being the most frequent approach (five procedures, 46%). Cardiopulmonary bypass was used in seven patients (87.5%). No early operative mortality was observed.

Conclusions

Pediatric coronary artery disease is heterogeneous and requires pathology-specific surgical strategies. Individualized surgical management was feasible with favorable early outcomes in a tertiary pediatric cardiac surgery center.

## Introduction

Anatomical or functional abnormalities of the coronary arteries in children can lead to impaired myocardial perfusion with serious consequences, including myocardial ischemia, heart failure, arrhythmias, and sudden cardiac death [1-3]. Unlike adult coronary artery disease, pediatric coronary artery disease is rare and represents only a small proportion of pediatric cardiac pathologies. Population-based data indicate that sudden cardiac death in children occurs at a rate of approximately 1.1 per 100,000 children per year, with cardiac diseases being a leading cause. Among these, coronary artery abnormalities represent an important though uncommon contributor to pediatric cardiac morbidity and mortality [4]. The rarity and heterogeneity of these conditions often pose diagnostic and therapeutic challenges [5,6].

Congenital coronary artery anomalies are an important subset of pediatric coronary diseases and include anomalies in coronary origin, course, and connections, including anomalous origin of a coronary artery from the pulmonary artery (e.g., anomalous left coronary artery from the pulmonary artery (ALCAPA)), absent coronary arteries, and abnormal connections such as coronary artery fistulae. These anomalies occur in less than 1% of the population and may present in isolation or in association with other congenital heart defects [1,5]. Acquired causes include coronary artery aneurysm, dilation, and thrombosis related to Kawasaki disease, which remains the leading cause of acquired coronary artery abnormalities in children, as well as rare causes such as trauma, infection, connective tissue disorders, and atherosclerosis [7,8].

Clinical manifestations in pediatric coronary artery disease are often nonspecific and may include fatigue, chest pain, shortness of breath, or symptoms of heart failure. Due to the heterogeneous presentations and rarity of these conditions, management strategies are highly individualized and often rely on multimodality imaging and specialized surgical expertise [1,5,9,10]. Despite the potential severity of pediatric coronary artery disease, published data remain limited, with most reports consisting of isolated case reports or small series focused on single anomaly types, with little emphasis on institutional experience or treatment outcomes in resource-limited settings, especially in Southeast Asia.

Therefore, this retrospective case series aims to describe the clinical characteristics, diagnostic evaluation, and surgical management of a heterogeneous spectrum of pediatric coronary artery pathologies treated at a tertiary pediatric cardiac surgery center between 2018 and 2025, providing insight into disease patterns and treatment strategies.

## Materials and methods

Study design and setting

This study was conducted as a single-center retrospective case series at a tertiary pediatric cardiac surgery center.

Study population

Eligible patients included all pediatric patients (<18 years of age) diagnosed with coronary artery disease who underwent surgical or catheter-based intervention at our institution between January 2018 and November 2025. Coronary artery disease was defined as structural or pathological abnormalities of the coronary arteries, including Kawasaki disease-related lesions, coronary artery fistula, anomalous coronary artery origin, and coronary abnormalities associated with congenital heart disease. Patients managed conservatively without surgical or interventional treatment, or those with insufficient clinical or operative data, were excluded.

Data collection

Clinical and operative data were obtained through retrospective review of institutional medical records and surgical databases. Extracted variables included demographic characteristics, clinical presentation, underlying etiology, coronary anatomy, operative details, perioperative parameters, and early postoperative outcomes.

Heart failure severity was assessed using the New York Heart Association (NYHA) functional classification, which was applied to evaluate symptom severity and exercise tolerance in this cohort of predominantly school-aged children and adolescents.

Given the retrospective nature of the study and the small sample size, data were summarized in aggregate form without individual patient identifiers.

Outcome measures

The primary objective was to describe clinical characteristics, surgical management, and early postoperative outcome, including in-hospital mortality and major perioperative complications. Long-term follow-up outcomes were not available for consistent analysis and were therefore not included in this study.

Statistical analysis

Descriptive statistics were used to summarize the data. Continuous variables were summarized as means, and categorical variables were reported as frequencies and percentages. No comparative or inferential statistical analyses were performed due to the descriptive nature of the study and limited sample size.

Ethical considerations

The study protocol was reviewed and approved by Cho Ray Hospital Ethics Committee (225-25/CN-HDDD). Given the retrospective design, the requirement for individual informed consent was waived. Patient confidentiality was maintained throughout the study.

Patient and public involvement

Patients and/or the public were not involved in the design, conduct, reporting, or dissemination of this retrospective study, which was based on review of medical records.

## Results

Patient characteristics

The baseline characteristics of the patients are summarized in Table 1. A total of eight pediatric patients were included in the study. There was an equal sex distribution, with four males and four females (50% each). The median age at presentation was 10 years.

**Table 1:** Baseline Characteristics of the Study Population (N = 8) BMI: body mass index

Variable	Value
Total number of patients	8
Age, median (IQR), years	10 (7–13.75)
BMI, median (IQR), kg/m²	15.5 (14–17)
Male sex, n (%)	4 (50%)
Female sex, n (%)	4 (50%)
Heart failure, n (%)	8 (100%)
Associated congenital heart disease, n (%)	2 (25%)

Clinical presentation

Presenting symptoms at admission are summarized in Table 2. Fatigue was the most common presenting symptom, reported in four patients (50%), followed by chest pain in two patients (25%) and cyanosis in two patients (25%). Clinical signs of heart failure were present in most patients, with NYHA class II symptoms in five patients (62.5%) and class III symptoms in three patients (37.5%) at the time of admission. 

**Table 2:** Presenting Symptoms at Admission NYHA: New York Heart Association

Symptom	n (%)
Fatigue	4 (50%)
Chest pain	2 (25%)
Cyanosis	2 (25%)
Heart failure (NYHA class II)	5 (62.5%)
Heart failure (NYHA class III)	3 (37.5%)

Etiology and coronary artery pathology

The etiologies and patterns of coronary artery involvement are presented in Table 3. The underlying coronary artery diseases were heterogeneous. Kawasaki disease-related coronary artery pathology, including aneurysm, dilation, or thrombosis, was the most frequent etiology, accounting for three patients (37.5%). Coronary artery fistula was identified in two patients (25%). Anomalous origin of the coronary artery was identified in three patients (37.5%), including one case of ALCAPA. In addition, two patients (25%) had coronary artery anomalies associated with tetralogy of Fallot. 

**Table 3:** Underlying Etiologies and Patterns of Coronary Artery Involvement †Among patients with anomalous coronary artery origin, one patient had anomalous left coronary artery from the pulmonary artery (ALCAPA).

Etiology & Coronary artery involvement	n (%)
Kawasaki disease–related aneurysm/dilation/thrombosis	3 (37.5%)
Coronary artery fistula	2 (25%)
Anomalous coronary artery origin (including ALCAPA)^ †^	3 (37.5%)
Coronary artery anomalies associated with tetralogy of Fallot	2 (25%)
Coronary artery involvement	n (%)
Right coronary artery (RCA)	4 (50%)
Left coronary artery (LCA)	3 (37.5%)
Left anterior descending artery (LAD)	3 (37.5%)

Regarding the anatomical distribution of coronary involvement, lesions most frequently affected the right coronary artery (RCA), which was involved in four patients (50%), followed by the left coronary artery (LCA) and the left anterior descending artery (LAD), each involved in three patients (37.5%). 

Surgical management

Surgical procedures and operative characteristics are summarized in Table 4. Eleven surgical procedures were performed in eight patients. Coronary artery bypass or coronary anastomosis procedures were the most frequently performed, accounting for five procedures (46%). Excision of abnormal coronary connections was performed in two procedures (18%), and complete repair of tetralogy of Fallot with avoidance of coronary injury was also performed in two procedures (18%). Use of cardiopulmonary bypass occurred in seven patients (87.5%), and aortic cross-clamping was required in six patients (75%). The mean operative time was six hours, the mean intensive care unit stay was 7.6 days, and the mean total hospital duration was 25 days. 

**Table 4:** Surgical Procedures and Operative Characteristics

Variable	n (%)
Total number of patients	8
Total number of surgical procedures	11
Coronary artery bypass/anastomosis	5 (46%)
Coronary artery reimplantation	1 (9%)
Coronary artery reconstruction/plasty	1 (9%)
Excision of abnormal coronary connection	2 (18%)
Complete repair of tetralogy of Fallot	2 (18%)
Use of cardiopulmonary bypass (CPB)	7 (87.5%)
Use of aortic cross-clamp	6 (75%)
Early operative mortality	0 (0%)

Early postoperative outcomes and complications

Early postoperative outcomes were generally favorable, as shown in Table 5. No early postoperative mortality was observed. Postoperative complications included pleural effusion in two patients, pericardial effusion in one patient, and postoperative pneumonia in one patient. Extracorporeal membrane oxygenation (ECMO) support was required in one patient. 

**Table 5:** Postoperative Complications ECMO: extracorporeal membrane oxygenation

Complication	n (%)
Pericardial effusion	1 (12.5%)
Pleural effusion	2 (25%)
Pneumonia	1 (12.5%)
ECMO support	1 (12.5%)
Mortality	0 (0%)

The mean operative duration was six hours. The mean length of stay in the intensive care unit was 7.6 days, and the mean total hospital length of stay was 25 days, as summarized in Table 6. 

**Table 6:** Perioperative Time

Time parameter	Mean
Operative time (hours)	6
Intensive care unit stay (days)	7.6
Total hospital stay (days)	25

## Discussion

In our study, acquired coronary artery pathology related to Kawasaki disease represented the most clinically significant subgroup. Notably, no cases of atherosclerotic coronary artery disease or familial hypercholesterolemia-associated coronary lesions were observed, highlighting the distinct etiologic spectrum of pediatric coronary pathology in our cohort. This finding is consistent with established knowledge that atherosclerosis is exceedingly rare in children and that Kawasaki disease remains the predominant cause of acquired coronary artery abnormalities in the pediatric population [8,11]. Coronary artery fistula and anomalous coronary artery origin were also frequently observed, reflecting the broad spectrum of congenital coronary anomalies described in pediatric populations [1,5]. Werner et al. reported that coronary artery anomalies are most commonly isolated lesions, but may occasionally be associated with other congenital heart defects, such as tetralogy of Fallot, transposition of the great arteries, pulmonary atresia, and common arterial trunk [12]. Consistent with these observations, two patients (25%) in our study had coronary artery abnormalities associated with tetralogy of Fallot.

Clinical presentation in pediatric coronary artery disease is often nonspecific. The findings are in line with previous reports demonstrating that symptoms may be subtle or atypical in children, leading to delayed diagnosis unless a high index of suspicion is maintained. This underscores the importance of comprehensive cardiac imaging, including echocardiography and advanced cross-sectional imaging, to accurately define coronary anatomy and guide management [1,5,9,12].

A range of operative techniques was employed in this highly individualized study, determined by coronary anatomy and associated cardiac lesions. Such tailored approaches are consistent with contemporary surgical principles in pediatric coronary artery disease, as no single strategy is applicable across the wide spectrum of coronary anomalies [13,14]. The frequent use of cardiopulmonary bypass and aortic cross-clamping in our series reflects the technical complexity of these procedures. In a multicenter cohort of 18 pediatric patients with a mean age of 12 months, coronary artery bypass grafting (CABG) was associated with an early mortality rate of 11.1%, and one patient with Kawasaki disease required heart transplantation one year after CABG [15]. In contrast, patients in our center were substantially older at the time of surgery, with a mean age of 10.1 years, and no early operative mortality was observed. CABG accounted for 46% of surgical procedures, representing the most frequently employed operative strategy. Differences in patient age, coronary artery size, and timing of intervention may contribute to variations in surgical approach and early outcomes between cohorts.

Taken together, the findings from this retrospective case series highlight that surgical management of pediatric coronary artery disease requires an individualized approach, taking into account patient age, coronary anatomy, underlying etiology, and associated cardiac lesions. Given the rarity of pediatric coronary artery disease, this case series provides valuable real-world clinical and surgical insights from a tertiary referral center, particularly in a resource-limited setting where published data remain limited.

Limitations

This study has several limitations. First, its retrospective design and small sample size may introduce selection bias and limit the generalizability of the findings. Second, due to variability in clinical documentation over the study period, standardized diagnostic parameters, imaging metrics, and detailed operative variables were not consistently available for all patients. In addition, the heterogeneity of underlying coronary pathologies and the absence of long-term follow-up limit the ability to draw definitive conclusions regarding long-term outcomes. Therefore, the findings should be interpreted as descriptive institutional experience rather than definitive evidence of treatment efficacy. 

## Conclusions

Pediatric coronary artery disease represents a rare and heterogeneous group of conditions with potentially severe clinical consequences. In this retrospective case series from a tertiary pediatric cardiac surgery center in a resource-limited setting, both congenital and acquired coronary artery pathologies were encountered. Our experience demonstrates that individualized surgical strategies can be performed safely with favorable early outcomes, even in environments with limited resources. These findings provide valuable real-world insight into the management of pediatric coronary artery disease in regions where published data remain scarce. Given the complexity and rarity of these conditions, advanced pediatric cardiac imaging and specialized expertise in ischemia assessment are essential for accurate diagnosis and surgical planning. Further multicenter studies incorporating multimodality imaging and long-term follow-up are warranted to refine optimal treatment strategies and assess long-term outcomes.

## References

[1] Brothers JA, Frommelt MA, Jaquiss RD, Myerburg RJ, Fraser CD Jr, Tweddell JS (2017). Expert consensus guidelines: anomalous aortic origin of a coronary artery. J Thorac Cardiovasc Surg.

[2] Cheitlin MD, De Castro CM, McAllister HA (1974). Sudden death as a complication of anomalous left coronary origin from the anterior sinus of Valsalva, a not-so-minor congenital anomaly. Circulation.

[3] Wu S, Fares M, Zellers TM, Jyothinagaram M, Reddy SR (2023). Diagnosis and management of congenital coronary artery fistulas in infants and children. Curr Cardiol Rep.

[4] Winkel BG, Risgaard B, Sadjadieh G, Bundgaard H, Haunsø S, Tfelt-Hansen J (2014). Sudden cardiac death in children (1-18 years): symptoms and causes of death in a nationwide setting. Eur Heart J.

[5] Feng J, Zhao J, Li J, Sun Z, Li Q (2023). Classification, diagnosis and clinical strategy of congenital coronary artery disease in children. Front Pediatr.

[6] Schleiger A, Kramer P, Dreysse S (2022). Coronary interventions in pediatric congenital heart disease. Pediatr Cardiol.

[7] McCrindle BW, Rowley AH, Newburger JW (2017). Diagnosis, treatment, and long-term management of Kawasaki disease: a scientific statement for health professionals from the American Heart Association. Circulation.

[8] de Ferranti SD, Steinberger J, Ameduri R (2019). Cardiovascular risk reduction in high-risk pediatric patients: a scientific statement from the American Heart Association. Circulation.

[9] Goo HW (2015). Coronary artery imaging in children. Korean J Radiol.

[10] Said SM, Dearani JA, Burkhart HM, Schaff HV (2010). Surgical management of congenital coronary arterial anomalies in adults. Cardiol Young.

[11] Krishnamurthy R, Suman G, Chan SS (2023). ACR Appropriateness Criteria®. Congenital or acquired heart disease. J Am Coll Radiol.

[12] Werner B, Wróblewska-Kałuzewska M, Pleskot M, Tarnowska A, Potocka K (2001). Anomalies of the coronary arteries in children. Med Sci Monit.

[13] Dodge-Khatami A, Mavroudis C, Backer CL (2002). Anomalous origin of the left coronary artery from the pulmonary artery: collective review of surgical therapy. Ann Thoracic Surg.

[14] Davies JE, Burkhart HM, Dearani JA (2009). Surgical management of anomalous aortic origin of a coronary artery. Ann Thorac Surg.

[15] Legendre A, Chantepie A, Belli E (2010). Outcome of coronary artery bypass grafting performed in young children. J Thorac Cardiovasc Surg.

